# Ionizing radiation enhances IL-6 and IL-8 production by human endothelial cells

**DOI:** 10.1080/09629359791677

**Published:** 1997-06

**Authors:** A. Van Der Meeren, J.-M. Bertho, M. Vandamme, M.-H. Gaugler

**Affiliations:** 1 Institut de Protection et de Sûreté Nucléaire Département de Protection de la santé de l'Homme et de Dosimétrie Section Autonome de Radiobiologie Appliquée à la Médecine IPSN, BP n ˚6, Fontenay-aux-Roses Cédex F-92265 France

## Abstract

Irradiation exposure is known to induce an inflammatory reaction. Endothelial cells play a crucial role both in the inflammatory process and in radiation damage. Therefore, supernatants and cell lysates of ^60^Co-irradiated human umbilical vein endothelial cells (HUVEC) have been assessed for the presence of pro-inflammatory cytokines. After gamma irradiation, interleukin (IL)-1α, IL-1β and tumor necrosis factor (TNF)-α remained undetectable in both cell supernatants and cell lysates. However, a dose-dependent increase in the production of IL-6 and IL-8 has been demonstrated up to 6 days after exposure. These data indicate that the pro-inflammatory cytokines IL-6 and IL-8 may be involved in the inflammatory response of vascular endothelium induced by exposure to ionizing radiation.


Mediators of Inflammation, **6**, 185–193 (1997)

### Ionizing radiation enhances IL-6 and IL-8 production by human endothelial cells

A. Van der Meeren[CA], J.-M. Bertho, M. Vandamme and M.-H. Gaugler

Institut de Protection et de Sûreté Nucléaire, Département de Protection de la santé de l'Homme et de Dosimétrie, Section Autonome de Radiobiologie Appliquée à la Médecine, IPSN, BP n°6, F-92265 Fontenay-aux-Roses Cédex, France

[CA]Corresponding Author
Tel: (+33) 1 46 54 95 49
Fax: (+33) 1 46 54 84 67

**Irradiation exposure is known to induce an inflammatory reaction. Endothelial cells play a crucial role both in the inflammatory process and in radiation damage. Therefore, supernatants and cell lysates of $^{60}$Co-irradiated human umbilical vein endothelial cells (HUVEC) have been assessed for the presence of pro-inflammatory cytokines. After γ irradiation, interleukin (IL)-1α, IL-1β and tumor necrosis factor (TNF)-α remained undetectable in both cell supernatants and cell lysates. However, a dose-dependent increase in the production of IL-6 and IL-8 has been demonstrated up to 6 days after exposure. These data indicate that the pro-inflammatory cytokines IL-6 and IL-8 may be involved in the inflammatory response of vascular endothelium induced by exposure to ionizing radiation.**

**Key words:** Cytokines, Endothelial cells, Inflammatory response, γ Irradiation

### Introduction

The inflammatory process is a classical pathophysiological response to ionizing radiation. Inflammatory lesions have been observed locally in a number of tissues, such as skin, intestine or lung and in a wide range of doses (between 5 and 40 Gy). Recent studies have shown early changes such as an increase in the number of adherent and emigrated leukocytes.[1–3] An early and persistent cytokine production following local exposure of rat intestine[4] or lung[5] has been observed and related to the late appearance of fibrosis.

In addition to these local effects, different investigators have shown the release of pro-inflammatory cytokines in peripheral blood. In humans, a total body irradiation of 10 Gy results in a rapid increase in the serum levels of TNF-α and IL-6, with a maximum reached 4 h after radiation exposure.[6] These increases were substantial but transient, and returned to baseline levels 24 h after irradiation. Circulating IL-1 and IL-6 have also been measured shortly after exposure to UV radiation.[7,8] However, the cellular source of the cytokines was not determined. Exposure of baboons to mixed neutron–γ field irradiation resulted in an early and transient increase in the serum level of IL-6, followed in some cases by a secondary release of IL-1, IL-6 and IL-8 a few days before the animals died;[9,10] this suggests a fatal prognosis associated with inflammatory response.

The production of pro-inflammatory cytokines by irradiated cells has also been investigated *in vitro*. Elevated levels of TNF-α and IL-1 have been found after irradiation of various human or mammalian cells, such as alveolar macrophages or tumour cells.[11,12] X-ray exposure induced an overproduction of IL-6 by fibroblasts[13] and of both IL-6 and IL-8 by glioma cells.[14] Increase in IL-6 and IL-8 production has also been detected after UV exposure of keratinocytes.[15,16] In all cases, these increases occurred early and the authors did not investigate the effects of irradiation later than 72 h.

Endothelial cells (ECs) play a crucial role in the initiation, development and maintenance of the inflammatory response. During the inflammatory process, leukocytes accumulate in the damaged tissue after transendothelial migration mediated by a cascade of events involving pro-inflammatory cytokines, chemokines and adhesion molecules. The major mediators of these processes are IL-1 and TNF-α. They appear to be produced mainly by activated monocytes/macrophages and, in turn, activate both these cells and vascular endothelial cells. This activation involves different steps where adhesion molecules are expressed, and where chemoattractants are released so allowing the transendothelial migration of leukocytes into the underlying tissue.[17–19]

© 1997 Rapid Science Publishers

Both IL-6 and IL-8 are involved in the inflammatory response. They have been detected in the serum of patients with inflammatory disorders[20–22] and can be produced in response to various damaging stimuli such as bacterial or viral infections,[23,24] or burns.[25] IL-8 is an important inflammatory mediator. It is a potent chemoattractant primarily for neutrophils but also eosinophils and a subset of lymphocytes.[26,27] IL-6 is a pleiotropic cytokine and substantial evidence exists about its inflammatory effects such as induction of acute phase proteins by the liver.[28] However, IL-6 has also been considered as an anti-inflammatory cytokine because of its ability to induce IL-1 and TNF-α antagonists.[29]

Evidence exists for the involvement of ECs in the radio-induced inflammatory response. An increase in leukocyte adhesion to irradiated endothelium has been observed *in vitro*, and is at least partly explained by the induction of some adhesion molecules, such as ICAM-1, in ECs from dermal microvasculature.[30,31] Data obtained by other investigators have shown the release of chemoattractants by irradiated bovine endothelial cells,[32,33] and suggest a biphasic response involving a lipid chemoattractant shortly after irradiation followed by the release of a protein attracting neutrophils. The chronic inflammatory lesions, commonly found after irradiation, could involve dysregulation of the vascular endothelium.[34]

The objective of this study was to investigate the *in vitro* radio-induced release of the pro-inflammatory cytokines IL-1α, IL-1β, TNF-α, IL-6 and IL-8 by HUVEC, to establish if these pro-inflammatory cytokines might be linked to radio-induced endothelial cell damage. Both time-course and dose–response studies have been carried out. Furthermore, since TNF-α has been shown to be released after radiation exposure, HUVEC have been irradiated both in the presence and in the absence of TNF-α. In addition, to advance the understanding of the mechanism of action of ionizing radiation, we investigated the possible role of intracellular oxidants in the regulation of cytokine production by HUVEC, and by using Northern blot analysis we determined the effect of ionizing radiation on cytokine gene expression.

### Materials and Methods

### Cell culture

HUVEC were obtained from the ATCC and routinely cultured in gelatin-coated flasks in F12K medium (Sigma, France) supplemented with 20% fetal calf serum (Gibco-BRL, France), endothelial cell growth supplement 60 μg/ml (Sigma, France), glutamine (Gibco-BRL, France), heparin 100 μg/ml (Sigma, France) and antibiotics. HUVEC are near-diploid non-transformed cells. In our culture conditions, cells reached confluency within 6 days. Cells were used between the sixth and the ninth passage after ATCC freezing.

For irradiation experiments, $1{\cdot}2 \times 10^5$ cells were plated on 35 mm diameter Petri dishes (Falcon, Becton-Dickinson, France) in the same medium as described above. Cells were activated with TNF-α 10 U/ml (specific activity $5 \times 10^7$ U/mg; R&D Systems, UK) and simultaneously irradiated 24 h after plating (control cells were neither activated with TNF-α nor irradiated). This concentration of TNF-α was chosen because it does not induce maximal production of IL-6 and IL-8. Supernatants were harvested and centrifuged to remove cell debris at various times after irradiation and frozen at −80°C until use. At each time point, the number of living adherent cells was evaluated using nigrosine exclusion after extensive washing of the cell monolayer. To evaluate the metabolic activity of irradiated cells, TNF-α was re-added to the 10 Gy-irradiated cells 6 days post-exposure, and cytokines were measured in the supernatant 48 h later.

To determine if the effect of γ irradiation could occur through the production of a soluble mediator by the irradiated cells, the following protocol was established: cells were irradiated 24 h after plating, and the supernatants were collected 72 h after irradiation. In parallel, cells were seeded according to the standard protocol, and 24 h after plating the medium was replaced by a 50:50 mixture of fresh medium/medium from the irradiated cells or the non-irradiated control cells. Concentrations of IL-6 and IL-8 were determined by ELISA of the cell supernatant 72 h later.

Cell lysates were performed as follows: after trypsinization, cell pellets were resuspended in 0.5% Triton X100, centrifuged $14\,000 \times g$ for 30 min, and then the supernatant was assessed for cytokine content.

### Irradiation

Proliferating cells were irradiated with a $^{60}Co$ source (ICO 4000) at a dose rate of approximately 1 Gy/min. The dose range tested was 1–20 Gy. The sham irradiated controls were treated under the same conditions.

### Antioxidant effect

We explored the role of oxidative stress in the response of HUVEC to ionizing radiation by the use of the antioxidant *N*-acetyl-L-cysteine (NAC, Sigma, France). Cells were treated with NAC (20 mM) 1 h before irradiation or TNF-α activation. After 72 h cell supernatants were collected and assessed for the presence of IL-6 and IL-8.

### Cytokine immunoassays

A double-antibody sandwich ELISA for the quantitative determination of the cytokines in cultured media was performed according to the manufacturer's recommendations (Amersham, France). The limits of detection were 3·9 pg/ml for IL-1α and β, 15·7 pg/ml for TNF-α, 3·13 pg/ml for IL-6 and 31·3 pg/ml for IL-8.

### Northern blot analysis

Total RNA was extracted according to the method of Chomczynski and Sacchi,[35] size-fractionated on a 1% formaldehyde–agarose gel (15 μg/lane), transferred to a Gene Screen nylon membrane (Dupont de Nemours, France) and fixed by heating (80°C, 3 h). Membranes were prehybridized for 3 h at 42°C and then hybridized overnight in hybridization buffer containing the human cDNA probes ($3 \times 10^6$ cpm/lane) labeled with $^{32}$P-αdCTP using the Megaprime DNA labelling system (Amersham, France). After hybridization, the membrane was washed. The quantification of radioactivity was made using the automated system InstantImager™ (Packard, France) after which the membranes were exposed to Hyperfilm-MP film (Amersham, France) at −80°C. IL-6 RNA expression was assessed using a 700 bp *Xmn*I–*Ban*I fragment and IL-8 using the R&D systems probe. Equal loading was analyzed by probing with a 1 kb *Hin*dIII fragment of the human glyceraldehyde-3-phosphate dehydrogenase (GAPDH, ATCC) cDNA cloned in the pBR322 vector.

### IL-6 bioassay

IL-6 activity was assessed using the 7TD1 cell line[36] obtained from ATCC. Briefly, cells were cultured in a 96 well-dish (1000 cells/well) in Dulbecco's modified medium (Gibco-BRL, France) supplemented with 10% fetal calf serum, glutamine, penicillin and streptomycin in the presence of serial dilutions of the supernatants to be tested or of human recombinant IL-6 (rhIL-6, R&D systems, UK; specific activity $1{\cdot}5 \times 10^6$ U/mg). The proliferative response of 7TD1 cells was assessed after 72 h of culture using the (3-4,5-dimethylthiazol-2-yl)-2,5-diphenyl-tetrazolium bromide colorimetric assay as described elsewhere.[37] One unit of IL-6 activity was defined as 50% of maximum proliferation obtained with rhIL-6. The detection limit of the test was 0·1 U/ml.

An IL-6 antibody (R&D Systems, England) was added to the culture medium containing the HUVEC supernatants at saturating concentration, in order to determine the specificity of the mitogenic activity.

### Statistical analysis

Statistical significance was calculated using the Mann–Whitney rank sum test. $P$ values $< 0.05$ were considered as significant.

### Results

### Effect of γ irradiation on endothelial cell growth

In order to assess the effect of γ irradiation on HUVEC growth, the viability and the number of cells were determined at various times after radiation exposure in the presence or absence of TNF-α (data not shown). Viability of the adherent cells was always found to be > 90%. However, a decrease in the number of cells was observed as early as 24 h post-exposure for the 5 and 10 Gy irradiated cells and 48 h for the 2 Gy irradiated cells. For example, 2 days and 6 days after irradiation, there were about 3-fold and 4.5-fold less cells respectively in the 10 Gy irradiated flasks than in the non-irradiated control flasks. The same observations were made with or without TNF-α. In addition, a noticeable change of the adherent cell morphology was observed; hypertrophic cells emerged by 2 days post-exposure.

### Effect of irradiation on pro-inflammatory cytokine production by HUVEC

IL-1α, IL-1β and TNF-α were detectable in neither the cell supernatant nor the cell lysate of control or γ-irradiated HUVEC cultures at any of the times tested.

Irradiation induced a higher production of IL-6 in both TNF-α stimulated and non-stimulated cells. This increase in IL-6 was both dose- and time-dependent (Fig. 1). The enhanced IL-6 production increased with the dose of irradiation, and the maximal effect was obtained later

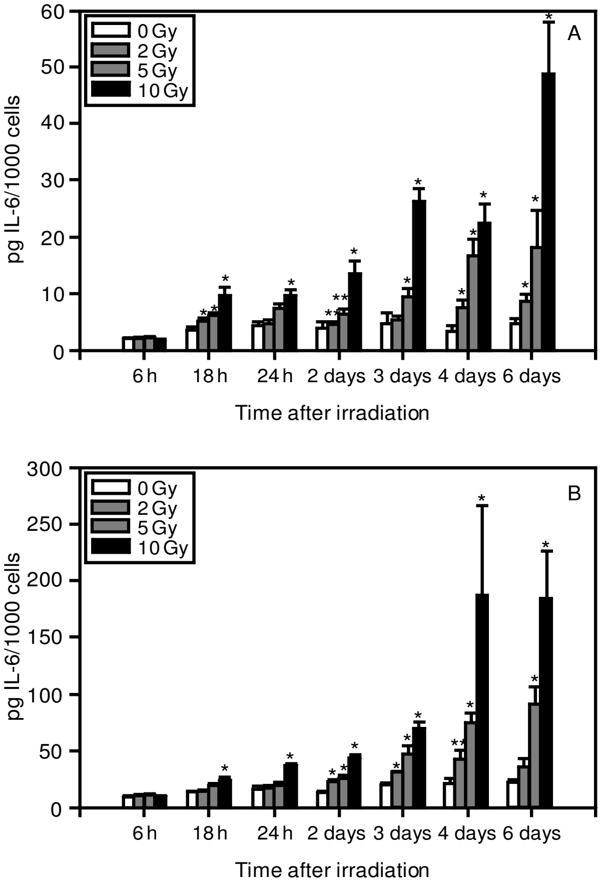

FIG. 1. Effect of γ irradiation on IL-6 production by HUVEC. Cells were irradiated 24 h after plating either in the absence (A) or in the presence (B) of TNF-α. Cell supernatants were collected from 6 h to 6 days after irradiation at doses of 2, 5, and 10 Gy. IL-6 determination was by ELISA. Results are expressed in pg/1000 viable cells. Values are the mean ± SEM of 2–4 separate experiments each performed in triplicate. (**, $P < 0.05$; *, $P < 0.01$)

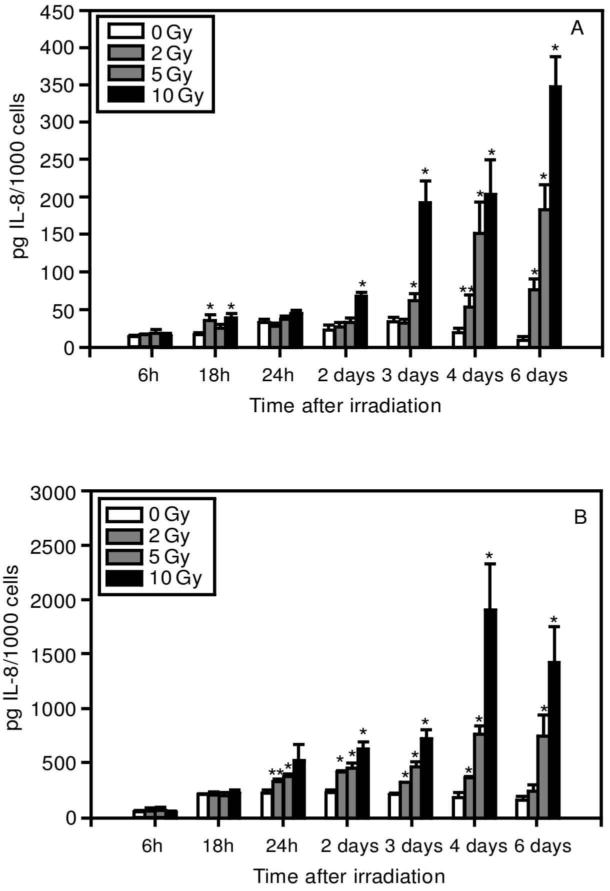

FIG. 2. Effect of γ irradiation on IL-8 production by HUVEC. Cells were irradiated 24 h after plating either in the absence (A) or in the presence (B) of TNF-α. Cell supernatants were collected from 6 h to 6 days after irradiation at doses of 2, 5, and 10 Gy. IL-8 determination was by ELISA. Results are expressed in pg/1000 viable cells. Values are the mean ± SEM of 2–4 separate experiments each performed in triplicate. (**, $P < 0.05$; *, $P < 0.01$)

with increasing doses: the plateau of IL-6 was reached within the first 24 h for the 2 Gy irradiated cells (no statistical difference was detected after that time) and 4 days post-exposure for the 5 Gy irradiated cells. No plateau was observed following a 10 Gy irradiation. Similar results were obtained when the cells were irradiated in the presence of TNF-α (Fig. 1B) except that the maximal production of IL-6 was detected 3 and 4 days after, respectively, a 5 Gy and 10 Gy exposure. Gamma exposure of HUVEC induced changes in the production of IL-8 similar to that noted for IL-6 (Fig. 2A and 2B). However, the effect of irradiation on IL-8 production was more pronounced than the effect on IL-6 production. The mean increase in IL-8 was 33.5-fold 6 days after irradiation whereas it was only 9.5-fold for IL-6. It is interesting to note that the effects of γ irradiation and TNF-α are synergistic. HUVEC irradiated at 10 Gy in the presence of TNF-α produced 6 days post-exposure, 136- and 36-fold more IL-8 and IL-6 respectively than the control cells.

To verify that the increased levels of IL-6 and IL-8 found in the cell supernatant of irradiated cells did not result from the release of the cytokine by damaged cells, we determined the amount of IL-6 and IL-8 present in the cell lysate for various times after irradiation. Table 1 shows the results obtained when cells were irradiated in the presence of TNF-α. Similar results were obtained in the absence of TNF-α except that IL-8 remained undetectable in the lysate of both irradiated and non-irradiated cells at all time periods tested. In the presence of TNF-α the amount of IL-6 and IL-8 present in the cell lysates remained at a level much lower than that in the supernatants. However, increases in the IL-6 and IL-8 levels were found in the cell lysates after γ irradiation, suggesting a *de novo* synthesis rather than a release of preformed protein.

**Table 1.** IL-6 and IL-8 production in the cell supernatant and cell lysate of HUVEC after γ irradiation in the presence of TNF-α

| Time after irradiation | γ Irradiation dose (Gy) | Pg IL-6/1000 cells | | Pg IL-8/1000 cells | |
|---|---|---|---|---|---|
| | | cell supernatant | cell lysate | cell supernatant | cell lysate |
| 6 h | 0 | 9.0 ± 3.7[a] | 1.1 ± 0.5 | 12.0 ± 3.3 | 4.4 ± 1.6 |
| | 2 | 11.1 ± 0.4 | 1.1 ± 0.2 | 12.9 ± 1.5 | 4.7 ± 1.1 |
| | 10 | 8.9 ± 1.1 | 1.3 ± 0.05 | 9.9 ± 1.3 | 4.3 ± 0.5 |
| 24 h | 0 | 13.4 ± 3.2 | 0.5 ± 0.1 | 278.0 ± 72.3 | 3.3 ± 0.2 |
| | 2 | 21.8 ± 2.7 | 0.6 ± 0.06 | 356.3 ± 51.5 | 5.9 ± 0.7 |
| | 10 | 41.7 ± 4.1 | 1.9 ± 0.5 | 1077.1 ± 283.3 | 10.2 ± 1.9 |
| 48 h | 0 | 16.6 ± 3.9 | 0.2 ± 0.05 | 266.8 ± 83.7 | 1.6 ± 0.2 |
| | 2 | 21.5 ± 3.8 | 0.2 ± 0.03 | 427.2 ± 34.3 | 2.5 ± 0.4 |
| | 10 | 46.0 ± 5.9 | 0.5 ± 0.2 | 759.2 ± 161.5 | 5.7 ± 1.6 |
| 96 h | 0 | 36.7 ± 10.0 | 0.1 ± 0.05 | 310.6 ± 85.1 | 0.7 ± 0.2 |
| | 2 | 60.2 ± 5.5 | 0.2 ± 0.02 | 477.3 ± 31.2 | 1.4 ± 0.4 |
| | 10 | 346.0 ± 50.0 | 2.2 ± 0.5 | 3747.4 ± 986.2 | 14.4 ± 5.1 |

[a]Values are mean ± SD of one representative experiment performed in triplicate.

Furthermore, the irradiated cells remained metabolically active; 6 days after a 10 Gy irradiation TNF-α restimulated the production of IL-6 and IL-8 by irradiated cells as efficiently as the non-irradiated cells (data not shown).

To determine if the effect of γ irradiation was a direct effect or occurred through induced production of a soluble mediator by the irradiated cells, we cultured non-irradiated cells in the presence of the supernatant from irradiated cells. No induction of IL-6 or IL-8 was observed after 72 h of culture as compared to the cells incubated with the supernatant of non-irradiated cells, suggesting that γ irradiation does not act through the release of a molecule into the cell supernatant (data not shown).

### Effect of N-Acetyl-cysteine (NAC) on IL-6 and IL-8 production

The use of an oxygen radical scavenger in HUVEC cultures had no effect on the radio-induced increase in production of IL-6 and IL-8, whereas the up-regulation of the two cytokines following TNF-α activation of irradiated or non-irradiated cells was partially inhibited (Table 2). A 2-fold decrease in IL-6 and IL-8 production was observed after NAC treatment of TNF-α activated cells. However, the antioxidant was more effective when the cells were both activated with TNF-α and irradiated with three times less IL-6 and IL-8 in the supernatants of NAC treated cells as compared with control cells.

**Table 2.** Effect of the antioxidant NAC on IL-6 and IL-8 production 72 h after TNF-α activation and/or 10 Gy irradiation

| | Pg IL-6/1000 cells | pg IL-8/1000 cells |
|---|---|---|
| Control | 2.7 ± 0.3[a] | 19.4 ± 1.8 |
| Control + NAC | 3.1 ± 0.3[NS b] | 17.33 ± 0.9[NS] |
| 10 Gy | 10.1 ± 1.8 | 57.0 ± 11.2 |
| 10 Gy + NAC | 8.8 ± 1.2[NS] | 32.5 ± 4.7[NS] |
| TNF-α | 42.4 ± 6.9 | 393.3 ± 76.4 |
| TNF-α + NAC | 21.7 ± 1.9[c] | 192.4 ± 37.3[c] |
| 10 Gy + TNF-α | 107.9 ± 21.4 | 745.3 ± 92.7 |
| 10 Gy + TNF-α + NAC | 35.5 ± 5.9[c] | 230.1 ± 21.1[d] |

[a]Values are mean ± SEM of three separate experiments performed in triplicate.
[b]For statistical analysis (Mann–Whitney test) the values were compared to the corresponding control without NAC. [NS], Not significantly different.
[c]$P < 0.05$.
[d]$P < 0.01$.

### Northern blot analysis

In order to determine if the irradiation effect occurred at the transcriptional level, we performed Northern blot analysis. HUVEC were either activated with TNF-α (10 U/ml) or irradiated (10 Gy). Although IL-6 and IL-8 transcripts were seen 4 h after TNF-α stimulation, they remained at a basal level up to 24 h after a 10 Gy irradiation (Fig. 3).

### Biological assays for IL-6 determination

The biological activity of IL-6 produced by HUVEC after irradiation was assessed using the IL-6 dependent cell line 7TD1. Fig. 4B represents the dose-response obtained after culture of 7TD1 cells for 72 h in the presence of supernatant obtained as described in Material and Methods; a dose dependent increase in IL-6 activity was observed. Furthermore, we obtained a strong correlation (r = 0.98) between biological activity determined by 7TD1 proliferation assay and the quantitative determination of IL-6 by ELISA (Fig. 4A). The specificity of the assay was determined by using monoclonal antibodies, which completely blocked the mitogenic activity of the HUVEC supernatants (data not shown). Thus, IL-6 produced by irradiated cells seems to be as active as the protein produced by non-irradiated cells.

### Discussion

In the present study, we investigated the involvement of inflammatory cytokines in the response of ECs *in vitro* to irradiation exposure. Our findings showed that IL-1α, IL-1β and TNF-α were not present in the supernatant of HUVEC irrespective of the culture and irradiation conditions, whereas IL-6 and IL-8 levels were increased up to 6 days after γ irradiation in a dose- and time-dependent manner. A 2 Gy

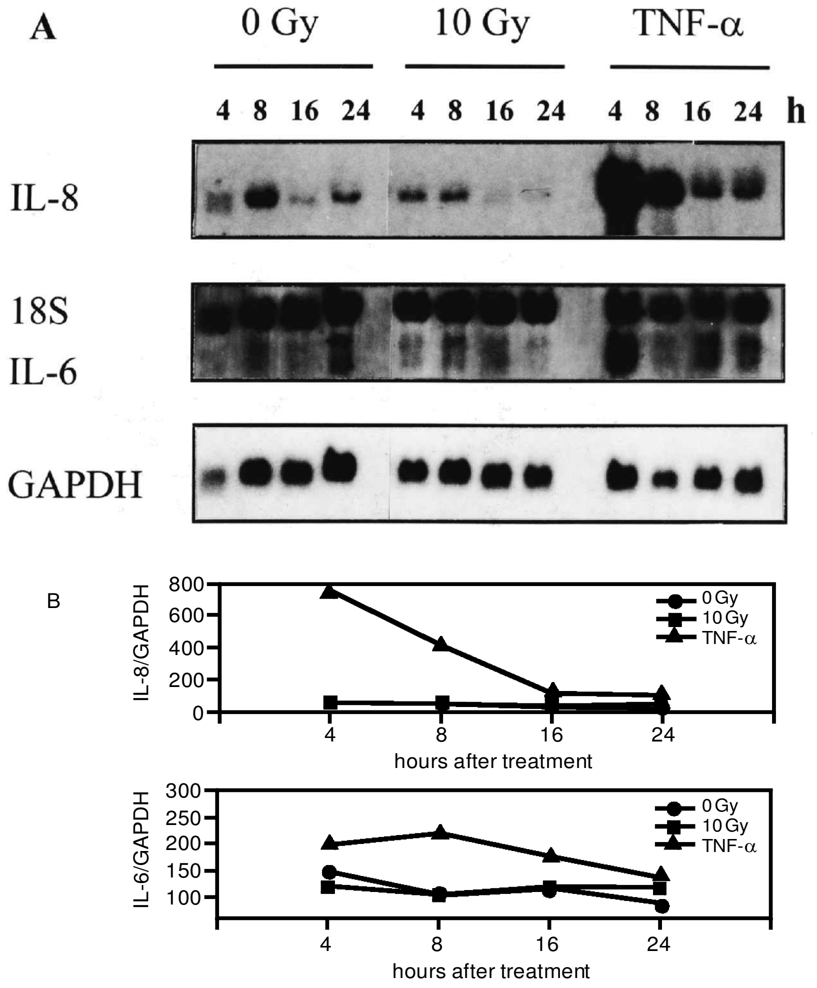

FIG. 3. Effect of TNF-α and 10 Gy-irradiation on IL-6 and IL-8 gene expression. RNA from control (0 Gy), irradiated (10 Gy) or TNF-α activated (TNF-α) cells was extracted 4, 8, 16 and 24 h after treatment and size fractionated and hybridized as described in Materials and Methods. (A) Autoradiographs. (B) The ratios IL-6/GAPDH and IL-8/GAPDH calculated after quantification of the radioactivity using an InstantImager.

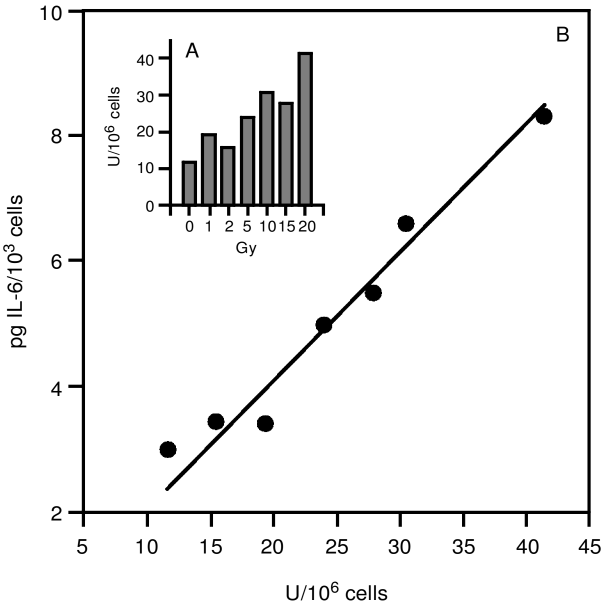

FIG. 4. Biological activity of IL-6 produced by irradiated HUVEC: correlation with ELISA determination. IL-6 bioactivity was measured by the 7TD1 proliferation assay as described in Materials and Methods. Cells were irradiated 24 h after plating at doses ranging from 1 to 20 Gy. The cell supernatant was collected 48 h after irradiation and used for the biological assay and ELISA determination. (A) IL-6 (U/ml) vs irradiation dose. (B) Correlation curve for the two assays: ordinate, IL-6 determined by proliferation assay; abscissa, IL-6 determined by ELISA.

irradiation is sufficient to induce a significant increase in cytokine production under some conditions. After a 10 Gy-irradiation, the enhancement of IL-6 and IL-8 production is progressive and persistent. It appears that this long-lasting effect is specific to γ exposure and is not a result of cell damage because the level of cytokines found in the cell lysates remains very low; this suggests that the release of cytokines from the cells could not contribute to the increased levels found after irradiation in the cell supernatant. As shown by others, TNF-α stimulated IL-6 and IL-8 production by HUVEC;[19] this up-regulation occurred early and reached a plateau within 2 days. In contrast, the cytokine-induced production following irradiation was progressive, and the maximum effect was not reached until 6 days after exposure to a dose of 10 Gy. Furthermore, our results demonstrate that not only IL-6 and IL-8 are induced by irradiation in the presence of TNF-α but also that the effects of TNF-α and γ irradiation are synergistic. Since TNF-α is likely to be present after irradiation, the synergistic phenomenon should also occur *in vivo*.

It has recently been shown that ionizing radiation can induce the transcription factor NFκB through a reactive oxygen intermediate signaling pathway.[38] Activation of NF-κB has also been involved in the overexpression of IL-6 observed after radiation exposure.[13] In addition, it has been demonstrated that treatment with hydroxyl radical scavengers decreased IL-8 production in different cellular models whereas it had no effect on IL-6.[39] TNF-α is known to stimulate the release of radical oxygen intermediates from a variety of cell lines.[40] Therefore, we tested the hypothesis that the increase in IL-6 and IL-8 production following TNF-α activation or radiation exposure of HUVEC would result from the increase in intracellular oxidants. Our results showed that treatment with NAC did not have any significant effect on the radio-induced production of IL-6 and IL-8 although it did result in a partial inhibition of the effect of TNF-α alone or associated with a 10 Gy-irradiation. This suggests that the synergy between TNF-α and γ rays involves an oxidant dependent pathway.

By Northern blot analysis we showed that TNF-α activation led to IL-6 and IL-8 up regulation whereas a 10 Gy exposure did not; this suggests that transcriptional activation is not the main regulatory mechanism of γ irradiation in HUVEC.

Different explanations of the radio-induced increase in IL-6 and IL-8 production may be proposed. Firstly, the increased production of these cytokines in the supernatants of irradiated cells could be the result of leakage from damaged cells. However, we demonstrated that the IL-6 and IL-8 content are significantly lower in the cell lysates compared to the supernatant. Secondly, the increase in IL-6 and IL-8 production could result from an indirect effect of ionizing radiation through the release of a molecule in the supernatant of irradiated cells. As discussed earlier, TNF-α and IL-1 are not responsible for such an effect, since none of these factors were found in either the cell supernatant or in the cell lysate. Furthermore, when non-irradiated cells were fed with supernatants obtained from irradiated cells, no increase in the production of IL-6 or IL-8 was observed. These data suggest a direct effect of γ irradiation on cytokine production. Finally, a decrease in the number of receptors present at the cell surface, a decrease in the release of the soluble form of the receptor, or a change in the affinity of the receptors could be induced by irradiation. This could explain the radio-induced increase in IL-6 production but not the increase in IL-8 because a recent study has shown the absence of IL-8 receptors at the surface of HUVEC[41] and, to our knowledge, no soluble form of the IL-8 receptor has yet been described. We determined that the soluble form of IL-6 receptor (IL-6sR) was not spontaneously released into the supernatant of HUVEC (data not shown), therefore, the increase in IL-6 cannot be explained by a decrease in IL-6sR. Further studies are necessary to evaluate the effect of irradiation on the expression of the membrane-bound component of the IL-6 receptor.

IL-6 and IL-8 may constitute the key effectors of the radio-induced response of endothelial cells. Through their multiple biological activities and in particular their respective induction of ICAM-1[42] and CD11b/CD18,[43] they may participate in the inflammatory reaction following ionizing radiation as described by Panès *et al.*[3] We have also observed the upregulation of ICAM-1 expression in HUVEC by γ irradiation in correlation with an increased adhesion of neutrophils on irradiated HUVEC (data not shown).

In conclusion, we have shown that IL-6 and IL-8 are involved in the inflammatory reaction of endothelial cells following γ irradiation, whereas TNF-α and IL-1 do not seem to be responsible for the inflammatory response. The increase in cytokine production was observed for doses as low as 2 Gy, and up to 6 days after exposure. Our results suggest the early and persistent effect of γ irradiation on the endothelium and the involvement of IL-6 and IL-8 in the development of the late phase of the radio-induced inflammatory response.

### References

1. Buell MG, Harding RK. Proinflammatory effects of local abdominal irradiation on rat gastrointestinal tract. *Dig Dis Sci* 1989; **34**: 390–399.
2. Fliss H. Ménard M. Rapid neutrophil accumulation and protein oxidation in irradiated rat lungs. *J Appl Physiol* 1994; **77**: 2727–2733.
3. Panès J, Anderson DC, Miyasaka M, Granger DN. Role of leukocyte–endothelial cell adhesion in radiation-induced microvascular dysfunction in rats. *Gastroenterology* 1995; **108**: 1761–1769.
4. Langberg CW, Hauer-Jensen M, Sung CC, Kane C. Expression of fibrogenic cytokines in rat small intestine after fractionated irradiation. *Radiother Oncol* 1994; **32**: 29–36.
5. Rubin P, Johnston CJ, Williams JP, Mcdonald S, Finkelstein JN. A perpetual cascade of cytokines postirradiation leads to pulmonary fibrosis. *Int J Radiat Oncol Biol Phys* 1995; **33**: 99–109.
6. Girinksy TA, Pallardy M, Comoy E, *et al.* Peripheral blood corticotropin-releasing factor, adrenocorticotropic hormone and cytokine (interleukin-1 beta, interleukin-6, tumor necrosis factor-alpha) levels after high- and low-dose total-body irradiation in humans. *Radiat Res* 1994; **139**: 360–363.
7. Konnikov N, Pincus SH, Dinarello CA. Elevated plasma Interleukin-1 levels in humans following ultraviolet light therapy for psoriasis. *J Invest Dermatol* 1989; **92**: 235–239.
8. Urbanski A, Schwarz T, Neuner P, *et al.* Ultraviolet light induces increased circulating Interleukin-6 in humans. *J Invest Dermatol* 1990; **94**: 808–811.
9. Agay D, Mestries JC, Martin S, Martin C, Hérodin F. L'inflammation radio-induite: implication de l'Interleukine 6. *Trav Scient SSA* 1992; **100**: 21–22.
10. Agay D, Veyret J, Van Uye A, Catérini R, Mestries JC. Influence d'un traitement par la r-hIL-6 associée ou non à l'AcSDKP sur la phase aiguë de l'inflammation radio-induite. *Trav Sci SSA* 1994; **15**: 19–20.
11. Hallahan DE, Spriggs DR, Beckett MA, Kufe DW, Weichselbaum RR. Increased Tumor Necrosis Factor α mRNA after cellular exposure to ionizing radiation. *Proc Natl Acad Sci USA* 1989; **86**: 10104–10107.
12. O'Brien-Ladner A, Nelson ME, Kimler BF, Wesselius LJ. Release of interleukin-1 by human alveolar macrophages after in vitro irradiation. *Radiat Res* 1993; **136**: 37–41.
13. Brach MA, Gruss HJ, Kaisho T, Asano Y, Hirano T, Herrmann F. Ionizing radiation induces expression of interleukin-6 by human fibroblasts involving activation of nuclear factor-kappa B. *J Biol Chem* 1993; **268**: 8466–8472.
14. Yamanaka R, Tanaka R, Yoshida S. Effects of irradiation on cytokine production in glioma cell lines. *Neurol Med Chir* 1993; **33**: 744–748.
15. Kirnbauer R, Köck A, Neumer P, *et al.* Regulation of epidermal cell Interleukin-6 production by UV light and corticosteroids. *J Invest Dermatol* 1991; **94**: 84–89.
16. Kondo S, Kono T, Sauder DN, McKenzie RC. IL-8 gene expression and production in human keratinocytes and their modulation by UVB. *J Invest Dermatol* 1993; **101**: 690–694.
17. Pober JS, Cotran RS. The role of endothelial cells in inflammation. *Transplantation* 1990; **50**: 537–544.
18. Mantovani A, Bussolino F, Dejana E. Cytokine regulation of endothelial cell function. *FASEB J* 1992; **6**: 2591–2599.
19. Swerlick RA, Lawley TJ. Role of microvascular endothelial cells in inflammation. *J Invest Dermatol* 1993; **100**: 111–115S.
20. Houssiau FA, Devogelaer JP, van Damme J, Nagant de Deuxchaisnes C, van Snick J. Interleukin-6 in synovial fluid and serum of patients with rheumatoid arthritis and other inflammatory arthritides. *Arthritis Rheumatism* 1988; **31**: 784–788.
21. Tanabe M, Ochi T, Tomita T, *et al.* Remarkable elevation of interleukin-6 and interleukin-8 levels in the bone marrow serum of patients with rheumatoid arthritis. *J Rheumatol* 1994; **21**: 830–835.
22. Brennan FM, Maini RN, Feldman M. Cytokine expression in chronic inflammatory disease. *Br Medical Bulletin* 1995; **51**: 368–384.
23. Hack CE, De Groot ER, Felt-Bersma JF, *et al.* Increased plasma levels of Interleukin-6 in sepsis. *Blood* 1989; **74**: 1704–1710.
24. Almeida GD, Porada CD, St Jeor S, Ascensao JL. Human cytomegalovirus alters interleukin-6 production by endothelial cells. *Blood* 1994; **83**: 370–376.
25. Ohzato H, Monden M, Yoshizaki K, *et al.* Systemic production of Interleukin-6 following inflammation. *Biochem Biophys Res Comm* 1993; **197**: 1556–1562.
26. Matsushima K, Baldwin ET, Mukaida N. Interleukin-8 and MCAF: Novel leukocyte recruitment and activating cytokines. In: Kishimoto T (ed) *Interleukins: Molecular Biology and Immunology.* Basel: Karger, 1992; 236–265.
27. Erger RA, Casale TB. Interleukin-8 is a potent mediator of eosinophil chemotaxis through endothelium and epithelium. *Am J Physiol* 1995; **12**: L117–122.
28. Kishimoto T, Akira S, Taga T. Interleukin-6 and its receptor: a paradigm for cytokines. *Science* 1992; **258**: 593–597.
29. Tilg H, Trehu E, Atkins MB, Dinarello CA, Mier JW. Interleukin-6 (IL-6) as an anti-inflammatory cytokine: induction of circulating IL-1 receptor antagonist and soluble Tumor Necrosis Factor receptor (p55). *Blood* 1994; **83**: 113–118.
30. Cornelius LA, Sepp N, Li LJ, *et al.* Selective up-regulation of Intercellular Adhesion Molecule-1 (ICAM-1) by Ultraviolet B in human dermal microvascular endothelial cells. *J Invest Dermatol* 1994; **103**: 23–28.
31. Heckmann M, Eberlein-Köning B, Wollenberg A, Przybilla B, Plewig G. Ultraviolet-A light radiation induces adhesion molecule expression on human dermal microvascular endothelial cells. *Br J Dermatol* 1994; **131**: 311–318.
32. Dunn MM, Drab EA, Rubin DB. Effects of irradiation on endothelial cell–polymorphonuclear leukocyte interactions. *J Appl Physiol* 1986; **60**: 1932–1937.
33. Matzner Y, Cohn M, Hyam E, *et al.* Generation of lipid neutrophil chemoattractant by irradiated bovine aortic endothelial cells. *J Immunol* 1988; **140**: 2681–2685.
34. Rezvani M, Hopewell JW, Robbins MEC. Initiation of non-neoplastic late effects: the role of endothelium and connective tissues. *Stem Cells* 1995; **13**: 248–256.
35. Chomczynski P, Sacchi N. Single-step method of RNA isolation by acid guanidium thiocyanate–phenol–chloroform extraction. *Analytical Biochem* 1987; **162**: 156–159.
36. Van Snick J, Cayphas S, Vink A, *et al.* Purification and NH2-terminal amino acid sequence of a T-cell-derived lymphokine with growth factor activity for B-cell hybridomas. *Proc Natl Acad Sci USA* 1986; **83**: 9679–9683.
37. Denisot F, Lang R. Rapid colorimetric assay for cell growth and survival. Modifications to the tetrazolium dye procedure giving improved sensitivity and reliability. *J Immunol Methods* 1986; **89**: 271–277.
38. Mohan N, Meltz ML. Induction of nuclear factor KB after low-dose ionizing radiation involves a reactive oxygen intermediate signaling pathway. *Radiat Res* 1994; **140**: 97–104.
39. Remick DG, Villarete L. Regulation of cytokine gene expression by reactive oxygen and reactive nitrogen intermediates. *J Leukoc Biol* 1996; **59**: 471–475
40. Gossart S, Cambon C, Orfila C, *et al.* Reactive Oxygen Intermediates as regulators of TNF-α production in rat lung inflammation induced by silica. *J Immunol* 1996; **156**: 1540–1548.
41. Petzelbauer P, Watson CA, Pfau SE, Pober JS. IL-8 and angiogenesis: evidence that human endothelial cells lack receptors and do not respond to IL-8 in vitro. *Cytokine* 1995; **7**: 267–272.

42. Caldenhoven E, Coffert P, Yuan J, Van de Stolpe A, Horn F, Kruijer W, van der Saag PT. Stimulation of the human intercellular adhesion molecule-1 promoter by interleukin-6 and interferon-γ involves binding of distinct factors to a palindromic response element. *J Biol Chem* 1994; **269**: 21146–21154.
43. Detmers PA, Lo SK, Olsen-Egbert E, Waltz A, Baggiolini M, Cohn ZA. Neutrophil-activating protein 1/interleukin-8 stimulates the binding activity of the leukocyte adhesion receptor CD11b/CD18 on human neutrophils. *J Exp Med* 1991; **171**: 1155–1162.

ACKNOWLEDGEMENTS. We thank Dr Patrick Gourmelon, Dr Laure Coulombel and Dr Nina Griffiths for their helpful discussions and Dr Jocelyne Aigueperse for her support. We also acknowledge Claire Squiban for her technical assistance.

**Received 14 January 1997; accepted in revised form 7 April 1997**
